# Chronic pain treatment in children and adolescents: less is good, more is sometimes better

**DOI:** 10.1186/1471-2431-14-262

**Published:** 2014-10-13

**Authors:** Tanja Hechler, Julia Wager, Boris Zernikow

**Affiliations:** German Paediatric Pain Centre, Children’s and Adolescents’ Hospital, Datteln, Department of Children’s Pain Therapy and Paediatric Palliative Care, Witten/Herdecke University, Dr.-Friedrich-Steiner Str. 5, 45711 Datteln, Germany

**Keywords:** Interdisciplinary, Outpatient treatment, Intensive inpatient treatment, Paediatric chronic pain

## Abstract

**Background:**

In children with chronic pain, interdisciplinary outpatient and intensive inpatient treatment has been shown to improve pain intensity and disability. However, there are few systematic comparisons of outcomes of the two treatments. The present naturalistic study aimed to compare the clinical presentation and achieved changes at return in three outcome domains (pain intensity, disability, school absence) between a) outpatients vs. inpatients and b) patients who declined intensive inpatient treatment and completed outpatient treatment instead (decliners) vs. those who completed inpatient treatment (completers).

**Methods:**

The study compared treatment outcomes between n = 992 outpatients vs. n = 320 inpatients (Analysis A) who were treated at a tertiary treatment centre and returned for a return visit within a one-year interval. In Analysis B, treatment outcomes were compared between n = 67 decliners vs. n = 309 completers of inpatient treatment. The three outcome domains were compared by calculating standardized change scores and clinically significant changes.

**Results:**

In analysis A, outpatients and inpatients reported comparably low levels of pain intensity (NRS 0–10; mean = 4, *SD* = 2.7) and disability (Paediatric Pain Disability Index (PPDI: 12–60; mean = 24; *SD* = 10) at the return visit. Compared to outpatients, more inpatients achieved clinically significant changes in pain intensity (52% vs. 45%) and disability (46% vs. 31%). There were also significantly greater changes in disability in the inpatient group (change score _outpatients_ = 1.0; change score _inpatients_ = 1.4; *F*_(1,1138)_ = 12.6, *p =* .011). School absence was substantially reduced, with approximately 80% in each group attending school regularly. Analysis B showed that even though inpatient decliners achieved improvements in the outcome domains, they reported greater disability at the return visit (PPDI mean _decliners_ = 27, SD = 9.9; PPDI mean _completers_ = 24, SD = 10) because they had achieved fewer changes in disability (change score _decliners_ = 0.9; change score _completers_ = 1.4; F_(1.334)_ = 5.7, *p =* .017). In addition, less decliners than completers achieved clinically significant changes in disability (25% vs. 47%).

**Conclusions:**

Inpatient and outpatient treatments are able to elicit substantial changes in pain intensity, disability and school absence. The results highlight the necessity of intensive inpatient pain treatment for highly affected children, as children who declined inpatient treatment and were treated as outpatients did less well.

## Background

Highly disabling chronic pain is a frequent complaint in children, with consistent prevalence estimates of approximately five percent in Western countries [1]. This condition can cause severe impairments for the child and suffering for his/her family [2]. Costs are also exorbitant in paediatric chronic pain [3]. These children access a variety of healthcare services, including primary care physicians, radiological examinations and visits to the emergency department [3].

It is widely accepted that the treatment of children with a severe chronic pain problem requires a specialised interdisciplinary approach and the stratification of treatment intensity, depending on the child’s status [2], as either an interdisciplinary outpatient treatment [4, 5] or a more intensive interdisciplinary pain treatment provided in an inpatient or day-hospital setting [6–8]. Children referred to outpatient treatment are thought to be able to achieve the requested changes with a less intense therapeutic dose [4]. Typical criteria for the recommendation of intensive interdisciplinary pain treatment are the child’s pain severity, degree of disability, school absences, and failure to progress under less intensive treatments [8, 9]. Systematic studies into the validation of criteria for treatment assignment are lacking [10], and it is primarily up to the clinicians’ judgement whether children are assigned to one form of treatment or the other.

Uncontrolled and controlled studies have shown that children are able to improve significantly and in a long-term manner when they obtain one of the two treatments (outpatient or intensive interdisciplinary pain treatment) [4–6, 8, 11]. Hechler et al. [4] showed that at a 12-month follow-up, almost 70% of the children who obtained an interdisciplinary outpatient treatment were able to attend school regularly. Pain intensity, disability and inappropriate coping strategies were also significantly reduced. Similarly, Logan et al. [8] found clinical and statistical improvements at a median of 10 months of follow-up in pain intensity, disability, physical functioning, medication use and emotional functioning in a study of 56 children obtaining intensive interdisciplinary pain treatment. This finding has also been confirmed within a randomised-controlled trial [12] in which children with chronic pain were assigned to either intensive interdisciplinary pain treatment or to a waiting-list control group. The results at immediate follow-up showed that approximately 60% of the intervention group had a clinical improvement, compared to only 14% of the waiting-list control group.

The two forms of treatment, however, have rarely been compared systematically in terms of their ability to decrease pain-related symptoms. Simons et al. [13] compared immediate outcomes of 50 children enrolled in intensive interdisciplinary pain treatment to 50 children who pursued outpatient multidisciplinary treatment matched for gender, pain diagnosis and level of functional disability. In line with their hypotheses, children enrolled in the intensive interdisciplinary pain treatment had significantly larger improvements in functional disability, and pain-related fear. While this study provides initial evidence for greater immediate improvements following intensive interdisciplinary pain treatment, several questions remain unanswered: First, differences in long-term outcome between the two treatments have not yet been investigated. Second, Simons et al. [13] lack a comparison of self-reported pain intensity, one of the core outcome domains according to clinical recommendations [14]. Third, while the authors control for initial differences in the clinical presentation, little is known on treatment outcomes of children with similar clinical presentation enrolled to intensive interdisciplinary pain treatment but who decline the recommendation of the pain team and pursue outpatient multidisciplinary pain treatment, instead.

The present naturalistic practice-based study had two objectives. The first objective was to compare the characteristics and changes in outcome domains (pain intensity, disability, school absences) between children who received outpatient treatment (low end of treatment intensity) or intensive interdisciplinary pain treatment (high end of treatment intensity) at the time point when they returned to the treatment centre within a one-year interval. Based on previous studies, we expected to find a similar improvement status at the time point of return in both groups. However, the two groups were expected to differ in the achieved changes, with inpatients achieving greater changes than outpatients due to the greater treatment intensity. The second objective was to compare outcomes between two groups of children who were recommended intensive interdisciplinary pain treatment by the pain team: a group who declined intensive interdisciplinary pain treatment but completed outpatient treatment instead (decliners) and a group who completed intensive interdisciplinary pain treatment (completers). This approach enables a comparison of treatment outcomes of two comparable study populations who share similar characteristics but who pursue different treatment pathways.

We expected to find greater changes in completers compared to decliners.

## Methods

### Sample

**Figure 1:**
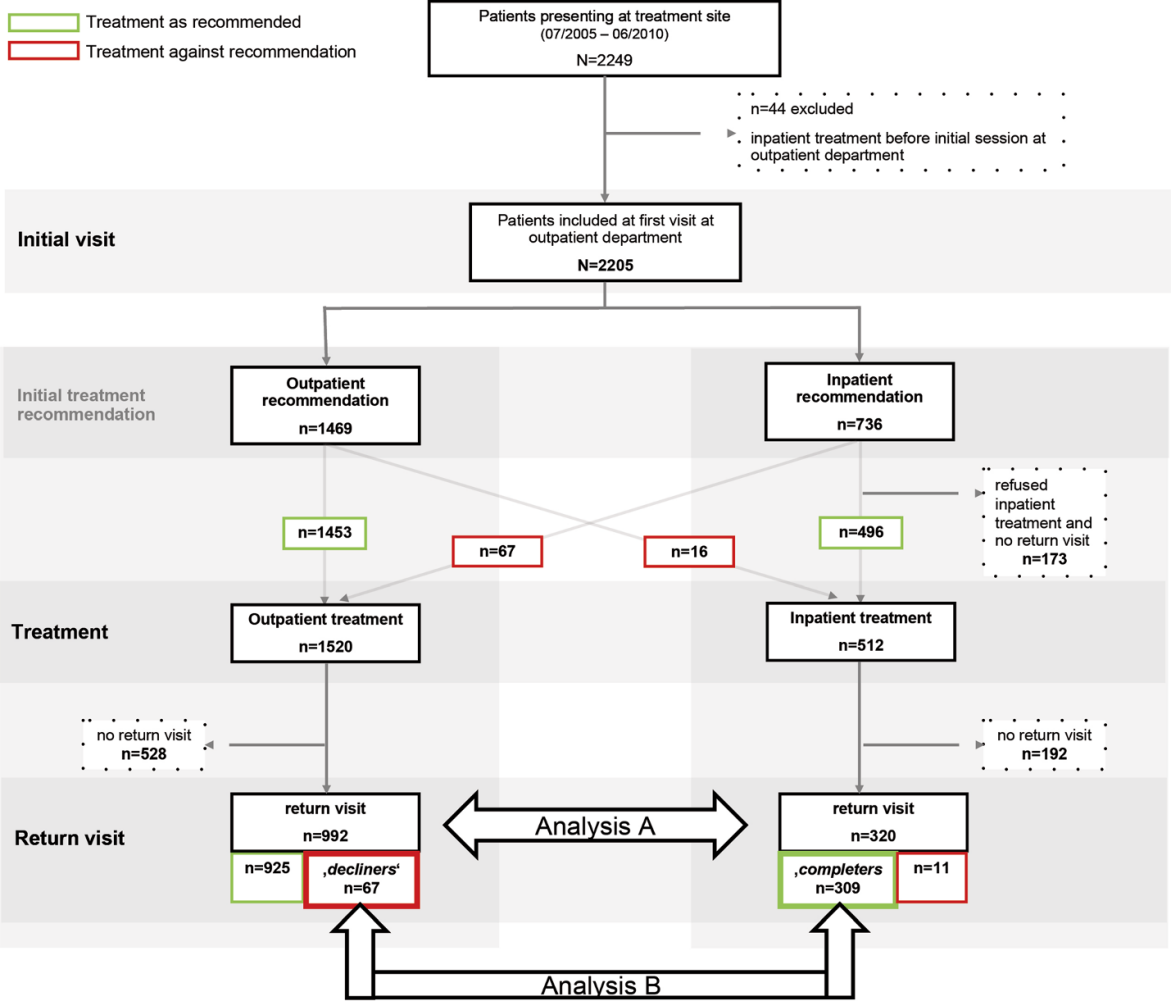
**Study flowchart and depiction of the two analyses of the study.**

The sample consisted of consecutive new children with chronic pain presenting at the German Paediatric Pain Centre from July 2005 to June 2010 who were treated as either outpatients or inpatients at the treatment centre (see Figure 1) and returned for a return visit within a one-year period. Detailed characteristics of these patients have been presented elsewhere [2]. Exclusion criteria were the following: pain treatment on other wards of the Children’s and Adolescents’ Hospital Datteln (e.g., on the gastroenterology ward) prior to the initial session or palliative diseases. Children with the latter were referred to the paediatric palliative service affiliated with the German Paediatric Pain Centre.

### Ethics

The present study was approved by the Ethics Committee of the Children’s Hospital in Datteln, Germany. All children and their parents provided written informed consent for data collection.

### General procedure at the German Paediatric Pain Centre

The German Paediatric Pain Centre offers a multimodal and interdisciplinary treatment within a stratified and stepped-care approach, consisting of outpatient or inpatient treatment. A paediatrician, a clinical child psychologist and a paediatric nurse evaluate the existing diagnostic information prior to the initial session and conduct the initial family session together. Each new referral is given an interdisciplinary 1.5-hour evaluation. The key goals of this evaluation are different dependent on the child’s clinical presentation.

#### Evaluation of the child’s clinical presentation as a core determinant for treatment allocation

The child’s clinical presentation was evaluated via standardised diagnostic tools such as the German Pain Questionnaire for Children and Adolescents (DSF-KJ) [15] and confirmed during the initial session. Referral to intensive interdisciplinary pain treatment rather than outpatient treatment was based on the following criteria [9, 12], of which the child had to fulfil at least three:

1. Severe pain-related disability assessed via the validated Pain-Disability Index (PPDI) [16] as scores > =36;
2. long pain duration of more than 6 months,
3. high average pain intensity (greater 6 on the NRS),
4. additional pain peaks (defined as pain intensity of 8 and above on the NRS),
5. regular school absence of at least 5 days within the preceding four weeks.

### Treatment at the German Paediatric Pain Centre

#### Outpatient treatment

This treatment comprises of an à priori evaluation of previous assessments and treatments of the child’s pain problem by the paediatrician (3–4 hours), the initial 1.5-hour session including different modules tailored to the particular needs of the individual patient, and a treatment plan comprising medical, and psychological treatment recommendations.

The key goals of the session are to identify the nature of the chronic pain experience, careful evaluation of present diagnostic findings, to educate the child and his or her parents on the biopsychosocial model of chronic pain, to provide strategies for pain relief, such as an adaptation of pain medication when necessary (67% were recommended pain medication during the initial session), teaching use of distraction techniques, change in parental focus on the child’s pain and strategies to attend school despite pain. These strategies and recommendations are summarised in the doctor’s letter, which is sent to the family and the primary paediatrician.

A follow-up appointment is scheduled for three months following the initial visit, but the children and their families are invited to return to the treatment centre whenever they feel that this might be necessary [4].

#### Intensive interdisciplinary pain treatment

For children with extremely high pain-related impairment (see criteria for referral), an intensive three-week multimodal inpatient program is recommended during the initial session [6, 17]. The key goals of the initial session are to enhance motivation for intensive interdisciplinary pain treatment by providing detailed information on the nature of the chronic pain condition, on the treatment program and on reasons for the intensive pain treatment. The session ends with a brief tour on the ward. The average waiting time to the inpatient unit is 3 to 6 weeks without any additional contact with the pain team in between [12].

An interdisciplinary team (paediatricians, clinical child psychologists, nursing and educational team (NET), pediatric psychiatrists, physiotherapists, art therapist, music therapist and social workers [9]) runs the inpatient program which consists of six modules: 1) information on the biopsychosocial concept of chronic pain and realistic goal attainment; 2) acquisition of pain-coping strategies, 3) treatment of related problems with school, peers or family; 4) teaching adequate parenting behaviour and family therapy; 5) optional interventions (pharmacological treatment (recommended to 30.6% (n = 98) following treatment), physiotherapy (recommended to 2.2% (n = 7) following treatment)). Pharmacological treatment is limited to pain due to inflammation or physical disease proven to be responsive to analgesics. Physiotherapy is used whenever advanced chronicity along with pronounced avoidance behaviour results in impaired functioning or impaired movement [9]. Physiotherapy is designed as an active therapy during which physical activity and active coping are enhanced. 6) Relapse prevention. Parents are actively engaged in the treatment as part of weekly family sessions and coaching sessions, during which the parents are taught to actively support their child and his or her engagement in healthy daily activities. Furthermore, reintegration into the child’s daily life is initiated from the second week onwards, which includes home visits and trips to their home school on one appointed day. A follow-up appointment is scheduled for three months following discharge, but the children and their families are invited to return to the treatment centre whenever they feel that this might be necessary (for a detailed description of the program, see [9]).

### Study procedure

All children eligible for the present study obtained the initial evaluation conducted by the interdisciplinary pain team. Following this, outpatients had no further contact with the pain team before the return visit. Inpatients were referred to the inpatient program with an average waiting time of 3 to 6 weeks. Inpatient treatment lasted for 3 weeks. For the present study, we assessed outcomes of the child at the time point of the first return to the treatment centre within a 12-months-period. The one-year period was chosen based on clinical experience. Children returning after 12 months usually present with a new pain problem. Hence, we defined the initial return visit to the treatment centre within a 12-months-period as the return visit under investigation. Return visits after a 12-months-period were considered as new referral and not included in the present analysis.

Data for the study were gathered retrospectively from clinical letters at the initial appointment and at return-visit. These letters included the pain diagnoses, treatment recommendations and a summary of the diagnostic set of questionnaires.

### Measures

*Average pain intensity* was reported as average pain intensity for the preceding four weeks on a numeric rating scale (NRS; 0 = no pain to 10 = maximal pain).

*Pain-related disability* in daily life was assessed via the validated German Paediatric Pain Disability Index (P-PDI) [16]. The questionnaire consists of 12 items (range 12–60) and has good internal consistency (Cronbach’s alpha = .87) and validity. It is used for children aged 11 years and above. Parents reported the disability for children aged younger than 11 years. We have previously found high agreement between self and parental report on pain-related disability (r = 0.624) [16].

*School absence* was assessed via parental report on the number of days missed at school within the preceding 20 school days for schoolchildren aged 6 years and above. A strong association has been shown between parental reports of school absence and official school attendance records [18]. Days of school missed were categorised into three categories to enhance communication of results: low (0–1 days missed), moderate (2–5 days missed), and high school absences (more than 5 days missed). The categories were derived from personal communication with the Federal Ministry of Education and with teaching staff, because normative data for categorising the severity of school absence is still lacking. These categories have been previously used (e.g., [2]).

### Statistical analyses

#### Group comparisons

*Analysis A*: Based on their completion of outpatient vs. inpatient treatment, we subdivided the children into outpatients and inpatients (see Flowchart Figure 1; Analysis A) and compared their treatment outcome at the first return visit to the treatment centre.

*Analysis B*: The second comparison of treatment outcome at the first return to the treatment centre made was between intensive inpatient treatment completers and decliners. The latter were children who deliberately refused intensive inpatient treatment but completed outpatient treatment instead (Analysis B, Figure 1). Of importance and in contrast to other health care systems, all patients in Germany have equal access to all levels of care. Hence, declining treatment was here framed as a willing decision of the child and his/her family.

### Statistical analysis for analysis A

Characteristics at the return visit were compared between outpatients and inpatients regarding days until return, sociodemographic characteristics, pain characteristics, disability and school absence. We computed *t-*Tests for independent samples, and Mann-Whitney *U*-test and *Chi*^*2*^-statistics to compare outpatients and inpatients. The effect sizes were computed and defined as follows: *d* for *t*-test (>.2 = small effect, >.5 = moderate effect, >.8 = large effect); *r* for *U*-test (>.1 = small; >.3 = medium; >.5 = large effect); and Cramer’s *V* for *Chi*^*2*^ Test (>.1 = small effect; >.3 = medium effect; >.5 = large effect) [19].

#### Differences in changes in pain intensity and disability between outpatients and inpatients

To explore individual changes in the metric outcome domains (pain intensity, pain-related disability), we computed standardised change scores by calculating the difference between the child’s scores at baseline and at follow-up and dividing them by the standard deviation (SD) of the group’s baseline score. Differences in these individual changes between inpatients and outpatients were calculated by an univariate analysis of variances (ANOVA), using the group as an independent variable (inpatients, outpatients) and the respective change scores as the dependent variables. Next, these differences were controlled for the influence of both the initial scores on the respective outcome domain and of the days until return (univariate analysis of covariances; ANCOVA). We controlled for the initial scores because we expected the inpatient group to report greater symptoms at the initial session. Controlling for days until return was performed because a longer time interval might be associated with greater changes in outcome domains. The reported effect size for these analyses was partial *eta*^*2*^ (>.01 = small; >.06 = medium; >.14 = large effect [19]). School absence constituted an ordinal variable with three school absence categories: low (0–1 day), moderate (2–5 days) and high (>5 days).

#### Differences between outpatients vs. inpatients in clinically significant changes in pain intensity and pain-related disability

To investigate whether the obtained changes in pain intensity and pain-related disability were equal, we determined the number of outpatients and inpatients with clinically significant changes in the two parameters, according to the study by Jacobson and Truax [20]. They suggested two criteria for a clinically relevant change: i) The magnitude of change between pre- and post-treatment scores should be statistically and reliably tested by use of a reliable change index (RCI). This resulted in three outcome stages: “no reliable change”; “reliable deterioration”; or “reliable improvement” for each patient and each parameter. ii) By the end of the treatment, the patients should move from a dysfunctional to a functional level to render them indistinguishable from healthy people. Therefore, cut-off points for the two parameters were defined. We adapted the procedure to define these cut-off points from prior publications [6, 11]: For P-PDI, a cut-off point of 23.09 (range: 12–60) was defined based on a previous study into the effectiveness of inpatient treatment [12]. For pain intensity, a raw-change of -1 on an NRS was used [21]. Hirschfeld et al. [21] recently showed within a group of 153 adolescents with severe chronic pain that raw changes of -1 NRS point can be considered as a minimally clinically significant difference. Using these cut-off points together with the RCI, we defined children with and without clinically significant changes in the two parameters. By use of Chi^2^-tests, we compared the number of outpatients vs. inpatients with clinically significant changes in the two parameters.

#### Comparison of changes in school absence

Changes in school absence were depicted in a cross table to investigate potential shifts from one school absence category at the first visit to another school absence category at the return visit. Differences between outpatients and inpatients in the distribution of children in the school absence categories were calculated separately for each of the three school absence categories at the initial visit by the use of Mann-Whitney *U*-tests.

### Statistical analysis for analysis B

We depicted how many children followed the recommendation of intensive inpatient treatment (completers) and how many refused the intensive inpatient treatment (decliners) but completed outpatient treatment instead (Figure 1). Characteristics at the return visit were compared between decliners and completers regarding days until return, sociodemographic characteristics, pain characteristics, disability and school absence, analogous to Analysis A. The differences in individual changes in pain intensity and pain-related disability, in clinically significant changes in pain intensity and pain-related disability, and in changes in the ordinal outcome of school absences were computed according to the statistical analyses described in Analysis A.

A two-tailed significance level of *p* = .05 was defined as significant. All analyses were calculated using SPSS 21.

## Results

### Return pattern of the children

From July 2005 to June 2010, 2249 children with chronic pain presented for treatment at the German Paediatric Pain Centre (see Zernikow et al. [2] for a detailed depiction of the sample). Of these children, 44 received inpatient treatment on other wards of the Children’s Hospital prior to the initial session at our institute and were excluded from further analyses. Of the remaining 2205 children, a total of 1312 children attended a return visit within a 12-months-period, including 992 outpatients and 320 inpatients (Figure 1). This sample constitutes the sample for Analysis A.

There were n = 736 children who were recommended inpatient treatment by the pain team. Additionally, n = 16 outpatients obtained inpatient treatment resulting in a total sample of n = 512. Of these, n = 320 returned to the treatment centre within 12 months. For Analysis B, we compared n = 67 children who declined inpatient treatment but pursued outpatient treatment instead to n = 309 inpatient completers.

Children who returned for treatment did not differ from those who did not return in age, sex, pain intensity or pain-related disability (*p* > .05). The two groups differed in the rate of school absence (*U* = 420,750; *Z* = -2.2; *p* = .026; *r* = |-.051|), showing higher rates of school absence in children who did not return.

### Analysis A: outpatients vs. inpatients

#### Comparison of characteristics of outpatients and inpatients at the time point of return

The characteristics of the two groups (inpatients, outpatients) at time point of return are depicted in Table 1. Inpatients returned significantly later compared to outpatients. They were also significantly older and more often female. The main pain locations also differed between the inpatients and outpatients. Headache was highly predominant in the outpatient group, followed by abdominal pain and musculoskeletal pain. In the inpatient group, headache was also the most frequent main pain location, but abdominal and musculoskeletal pain had a higher prevalence compared to outpatients. Pain intensity and pain-related disability did not differ between the groups when the patients came for a return visit. The average pain intensity was approximately four in both groups. School absence at the return visit was more frequent in former inpatients, with 22% reporting moderate or high school absence within the preceding four weeks compared to 16% of the outpatients.

**Table 1:** **Characteristics at return visit (outpatient vs. inpatient)**

	Outpatients (***n*** = 992)				Inpatients (***n*** = 320)				Statistics		
	Mean	***SD***	Range	***n***(%)	Mean	***SD***	Range	***n***(%)	Parameter ( ***t***, ***Chi*** ^***2***^)	***p***-value	Effect size ^§^
Days until return visit	97.0	38.1	12–344	992	128.6	47.4	29–313	320	***t*** **(1310) = 12.117**	**<.001**	***d*** **= 0.83**
Age	11.0	3.3	1–19	992	13.9	2.5	5–19	320	***t*** **(1310) = 14.234**	**<.001**	***d*** **= 0.88**
Sex									***Chi*** ^***2***^ **(1) = 14.125**	**<.001**	***V*** **= .104**
Male				421 (42)				98 (31)			
Female				571 (58)				222 (69)			
Main pain location									***Chi*** ^***2***^ **(3) = 128.2**	**<.001**	***V*** **= .313**
Head				813 (82)				168 (53)			
Abdomen				110 (11)				68 (21)			
Musculoskeletal				63 (6)				71 (22)			
Other				4 (0.4)				12 (4)			
Average pain intensity^$^	4.2	2.7	0–10	942	4.3	2.9	0–10	314	*t*(1254) = 0.546	.585	-
Pain-related disability^#^	24.8	10.7	12–60	894	23.9	10.0	12–60	298	*t*(1190) = -1.244	.214	-
School absence^&^			0–20						***U*** **= 184,632;** ***Z*** **= 12.2**	**<.001**	***r*** **= .357**
Low (0–1 day)				738 (84)				233 (78)			
Moderate (2–5 days)				117 (13)				40 (13)			
High (>5 days)				22 (3)				27 (9)			

^§^Effect sizes for *t*-tests = *d*; for *U*-Tests = *r*; and for *Chi* ^*2*^-test = Cramer’s *V*.

^$^Numeric rating scale (NRS) 0–10: 0 = no pain, 10 = worst pain.

^#^Paediatric Pain Disability Index (P-PDI [15], range 12–60).

^&^There were *n* = 66 children aged younger than six years for whom school absence could not be assessed.

Boldface data reflect significant differences between the two groups.

#### Differences in changes in pain intensity and disability between outpatients and inpatients

At the return visit, children in both groups achieved moderate to large changes in pain intensity (Table 2). The greatest change was found for disability. Generally, change scores at the return visit were larger in inpatients compared to outpatients (all *p* < .01). When controlled for initial scores and days until return, the two groups differed significantly in the change of pain-related disability. Specifically, inpatients reported greater changes in disability compared to outpatients.

**Table 2:** **Comparison of individual changes (pain intensity, pain-related disability) between inpatients and outpatients**

	Group						Statistics for main effect “group”							
	Outpatients			Inpatients			ANOVA				ANCOVA covariate: initial score + days until return			
***Individual changes*** ^***§***^	Mean	***SD***	***n***	Mean	***SD***	***n***	***df***	***F***	***p***-value	***eta*** ^***2***^	***df***	***F***	***p***-value	***eta*** ^***2***^
Pain intensity^$^	0.9	1.5	920	1.2	(1.5)	312	**1,1230**	**10.5**	**.001**	**.008**	1,1228	0.1	.728	-
Pain-related disability^#^	1.0	1.2	850	1.4	(1.2)	292	**1,1140**	**33.2**	**<.001**	**.028**	**1,1138**	**12.6**	**<.001**	**.011**

Note:

^§^Individual change: (Child’s score at baseline – child’s score at follow-up)/*SD* of the group baseline score; Interpretation of standardised change scores: 0.6 to 0.99 is considered a moderate change; ≥1.0, a large change.

^$^Numeric rating scale (NRS) 0–10.

^#^Paediatric Pain Disability Index (P-PDI [15]).

Boldface data reflect significant differences between the two groups.

#### Differences in clinically significant changes in pain intensity and disability between outpatients and inpatients

**Figure 2:**
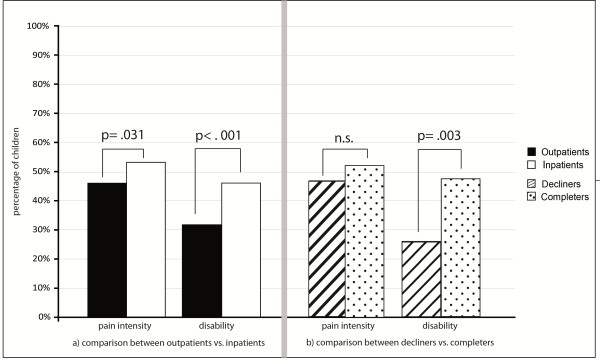
**Comparison between outpatients vs. inpatients and between decliners vs. completers regarding clinically significant changes in pain intensity and disability.** The figure shows the number of children with clinically significant changes in pain intensity and disability. The left part of the figure **(a)** shows the comparison between outpatients and inpatients. The right part **(b)** shows the comparison between decliners and completers. Clinically significant changes were defined according to Jacobson and Truax as i) statistical and reliable change between pre- and post-treatment scores, and ii) as patients’ move from a dysfunctional to a functional level. Cut-off for pain intensity was defined as a raw change of -1 on an NRS [21]. Cut-off for disability was defined as a PPDI-score of 23.09 (range: 12–60) based on previous studies [12].

More inpatients than outpatients achieved clinically significant changes in pain intensity (Chi^2^(1) = 4.629; p = .031; Cramer’s V = .061). Specifically, 52% (n = 162) of the inpatients compared to 45% (n = 413) of the outpatients achieved clinically significant changes in pain intensity (Figure 2).

Similarly, more inpatients than outpatients achieved clinically significant changes in pain-related disability (46%, n = 135 vs. 31%, n = 265) (Chi^2^(1) = 21.649; p < .001; Cramer’s V = .138).

#### Differences in changes in school absence between outpatients and inpatients

**Figure 3:**
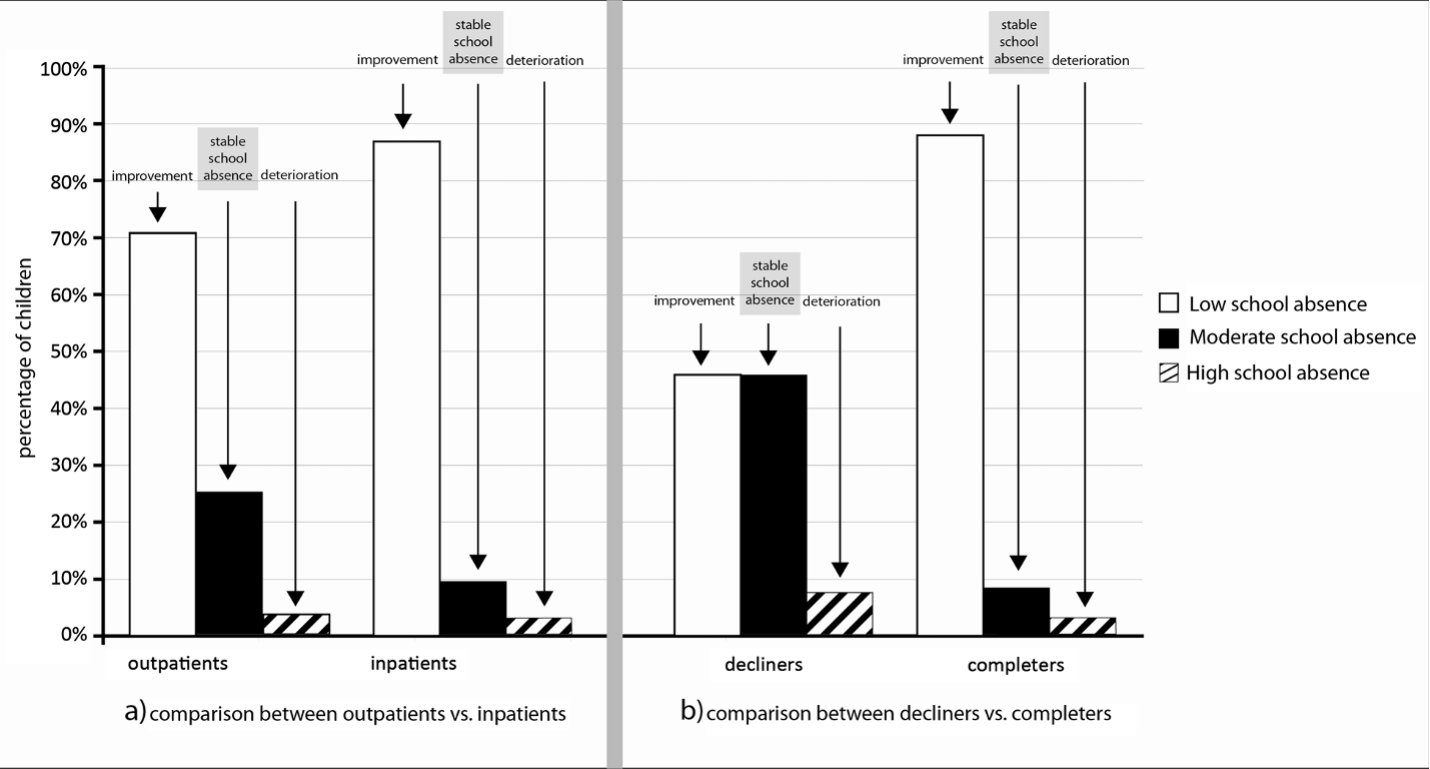
**Comparison between outpatients vs. inpatients and between decliners vs. completers regarding improvements, stable or deterioration in school absence for the group of children with initially moderate school absence.** The figure shows changes in school absence for children with initially moderate school absence (i.e., 2 to 5 days within four school weeks). The left part of the figure **(a)** shows the comparison between outpatients vs. inpatients. The right part **(b)** shows the comparison between decliners vs. completers. Children were assigned to the ‘Improvement-group’ if they reported low school absence (<2 days/week) at the return visit. ‘Stable school absence’ represents children who still reported moderate school absence (2 to 5 days/week) at the return-visit and ‘deterioration’ represents children who reported high school absence (>5 days/week) at the return-visit.

Table 3 depicts the changes in school absence from the initial visit to the return visit for the outpatient and inpatient groups.The two groups differed in their changes in school absence within the group that reported moderate school absence at the initial visit (Figure 3). Inpatients achieved greater changes.

**Table 3:** **Comparison of changes in school absence for inpatients and outpatients**

					Statistics			
		***School absence at return visit***				Mann-Whitney ***U***-Test		
***School absence*** ^***&***^ ***at initial visit***	Group	Low (0–1 days)	Moderate (2–5 days)	High (>5 days)	***U***	***Z***	***p***-value	***r***
**Low** (0–1 days)	Outpatients	454 (92.5)	34 (6.9)	3 (0.6)	17,792	-0.6	.524	-
	Inpatients	70 (94.6)	3 (4.1)	1 (1.4)				
**Moderate** (2–5 days)	**Outpatients**	**181 (71.3)**	**64 (25.2)**	**9 (3.5)**	**7,691**	**-2.7**	**.006**	**|-.151|**
	**Inpatients**	**63 (87.5)**	**7 (9.7)**	**2 (2.8)**				
**High** (>5 days)	Outpatients	69 (73.4)	16 (17.0)	9 (9.6)	6,988	1.8	.068	-
	Inpatients	83 (62.4)	28 (21.1)	22 (16.5)				

Notes: Frequencies are depicted as *n* (%).

^&^School absence is reported for children aged six years and older.

Boldface data reflect significant differences between the two groups.

### Analysis B: decliners of intensive inpatient treatment vs. completers

#### Comparison of characteristics of decliners vs. completers at the time point of return

The two groups did not differ regarding sex or main pain location. Children in both groups were on average 13 years old (SD_decliners_ = 3.2; SD_completers_ = 2.4). There was a significant age difference between the two groups (*t*(df = 374) = 1.99, *p <* .05) due to an outlying three years of age in the group of inpatient decliners. The difference disappeared when the outlier was excluded. Both groups also reported comparable levels of pain intensity at the return visit (Table 4). Decliners returned significantly earlier compared to completers. Pain-related disability was significantly higher in decliners, who also reported moderate school absences more frequently.

**Table 4:** **Characteristics at return visit (decliners vs. completers)**

	Inpatient treatment decliners (***n*** = 67)				Inpatient treatment completers (***n*** = 309)				Statistics		
	Mean	***SD***	Range	***n***(%)	Mean	***SD***	Range	***n***(%)	Parameter ( ***t***, ***Chi*** ^***2***^)	***p***-value	Effect size ^§^
Days until return visit	88.1	56.7	18–323	67	129.4	47.7	29–313	309	***t*** **(374) = 6.203**	**<.001**	***d*** **= 0.79**
Sex									*Chi* ^*2*^(1) = 0.22	.882	*-*
Male				21 (31)				94 (30)			
Female				46 (69)				215 (70)			
Main pain location									*Chi* ^*2*^(3) = 0.998	.802	*-*
Head				39 (59)				162 (53)			
Abdomen				13 (20)				66 (21)			
Musculoskeletal				12 (18)				68 (22)			
Other				2 (3)				12 (4)			
Average pain intensity^$^	4.8	2.8	0–10	63	4.4	2.9	0–10	304	*t*(365) = -1.056	.834	-
**Pain-related disability** ^**#**^	27.2	9.9	12–47	58	23.9	10.0	12–60	288	***t*** **(344) = -2.259**	**.024**	***d*** **= 0.33**
School absence^&^			0–20						***U*** **= 9,100;** ***Z*** **= 2.1**	**.032**	***r*** **= .12**
Low (0–1 day)				41 (71)				226 (78)			
Moderate (2–5 days)				12 (21)				36 (13)			
High (>5 days)				5 (9)				27 (9)			

^§^Effect sizes for t-tests = d; for *U*-Tests = r; and for *Chi* ^*2*^-test = Cramer’s *V*.

^$^Numeric rating scale (NRS) 0–10; 0 = no pain, 10 = worst pain.

^#^Paediatric Pain Disability Index (P-PDI, [15], range 12–60.

^&^School absence is reported for children aged six years and older.

Boldface data reflect significant differences between the two groups.

#### Differences in changes in pain intensity and disability between decliners vs. completers

Completers achieved greater changes in pain-related disability compared to decliners. This holds true, even after controlling for the initial score and time interval until return (Table 5). The achieved large changes in pain intensity were comparable between the two groups.

**Table 5:** **Comparison of standardised change scores between decliners vs. completers**

							Statistics for main effect group							
	Inpatient treatment decliners			Inpatient treatment completers			ANOVA				ANCOVA covariate: initial score + time difference			
***Individual changes*** ^***§***^	Mean	***SD***	***n***	Mean	***SD***	***n***	***df***	***F***	***p***-value	***eta*** ^***2***^	***df***	***F***	***p***-value	***eta*** ^***2***^
Pain intensity^$^	1.1	1.4	63	1.2	1.5	303	1,364	0.3	.573	-	1,362	0.7	.420	-
Pain-related disability^#^	0.9	1.0	56	1.4	1.2	282	**1,336**	**11.8**	**.001**	**.034**	**1,334**	**5.7**	**.018**	**.017**

Note:

^§^Individual change: (Child’s score at baseline – child’s score at follow-up)/*SD* of the group baseline score; Interpretation of change scores: 0.6 to 0.99: moderate change; ≥1.0 large change.

^$^Numeric rating scale (NRS) 0–10.

^#^Paediatric Pain Disability Index (P-PDI, [15]).

Boldface data reflect significant differences between the two groups.

#### Differences in clinically significant changes in pain intensity and disability between decliners vs. completers

A similar amount of inpatient decliners and inpatient completers, i.e. approximately half in each group (decliners: n = 29, 46%; completers: n = 156, 52%) achieved clinically significant changes in pain intensity (Chi^2^(1) = 0.621; p = .431) (Figure 2).

More completers than decliners achieved clinically significant changes in pain-related disability. Specifically, there were 47% (n = 132) of the completers compared to 25% (n = 14) of the decliners with clinically significant changes in pain-related disability (Chi^2^(1) = 9.056; p = .003; Cramer’s V = .164).

#### Differences in changes in school absence between decliners vs. completers

The changes in school absence for decliners vs. completers are depicted in Table 6.

**Table 6:** **Comparison of changes in school absence between decliners and completers**

					Statistics			
		***School absences at return visit***			Mann-Whitney ***U***-Test			
***School absences*** ^***&***^ ***at initial visit***		Low (0–1 days)	Moderate (2–5 days)	High (>5 days)	***U***	***Z***	***p***-value	***r***
**Low** (0–1 days)	decliners	20 (95.2)	1 (4.8)	0	742	0.2	.858	-
	completers	66 (94.3)	3 (4.3)	1 (1.4)				
**Moderate (2–5 days)**	**decliners**	**6 (46.2)**	**6 (46.2)**	**1 (7.7)**	**265**	-3.6**	**<.001**	**|-.395|**
	**completers**	**62 (88.6)**	**6 (8.6)**	**2 (2.9)**				
**High** (>5 days)	decliners	11 (61.1)	4 (22.2)	3 (16.7)	1252	-0.3	.902	-
	completers	82 (63.1)	26 (20.0)	22 (16.9)				

Note: Frequencies are depicted as *n* (%).

^&^School absence is reported for children aged six years and older.

Boldface data reflect significant differences between the two groups.

The two groups differed in their change in school absences within the group with initially moderate school absences (Figure 3). Completers with initially moderate school absence achieved greater changes in school absence than decliners (U = 265; Z = -3.6; *p <* .001) with more than 88% of the completers compared to 46% of the decliners reporting low school absence.

The majority of both, decliners and completers, with initially low school absence reported low school absence at the return-visit (U = 742; Z = 0.179; *p* = .858). Similarly, approximately 60% of both, decliners and completers with initially high school absence reported low school absence, 20% reported moderate and 16% reported high school absence at the return-visit (U = 1,1152; Z = -0,123; *p* = .902).

## Discussion

The present study aimed to compare changes in three outcome domains between children obtaining interdisciplinary outpatient treatment and children obtaining intensive interdisciplinary inpatient treatment and between decliners and completers of inpatient treatment. Overall, the results indicate that both treatments are effective in improving pain intensity, disability and school absence, in line with previous effectiveness studies [4, 5, 11, 12]. The present results, however, suggest that substantially greater changes can be achieved via intensive inpatient treatment, in particular with regards to pain-related disability and school absence.

In line with our hypothesis, children in intensive inpatient treatment achieved greater changes in pain intensity, pain-related disability and in school absence. There were also significantly more inpatients with clinically significant changes in pain intensity (52% vs. 45%) and disability (46% vs. 31%). These results highlight the potential of intensive interdisciplinary pain treatment to achieve significant and clinically relevant improvements. The change in school absence is particularly important. First, results suggest that outpatient and inpatient treatment enables children to maintain regular school attendance. Second, results suggest that in both groups high school absence can be substantially reduced as reflected by an incidence of less than 10% of children with high school absence at the return-visit in each group. This also means that approximately half of the inpatient sample that initially reported high school absence is now able to attend school. Third, for children who initially reported moderate school absence (approximately 30% in each group), results suggest that intensive interdisciplinary pain treatment can result in more pronounced decreases of school absence. Potential candidates for these greater changes after intensive inpatient treatment can be the treatment intensity, daily treatment with various professionals, specific school-based interventions, such as attending home-school during inpatient treatment [9] and a more pronounced decline in pain-related fear during intensive pain treatment [13].

Despite these positive findings, there was a group of less than 10% of the children who had obtained intensive inpatient treatment that maintained a high level of school absence at return. This is in line with previous effectiveness studies for intensive inpatient treatment, which reported a percentage of approximately 10 to 20% with negative treatment results [11]. For this particular group, it is important to identify reasons for the stable high school absence, such as stable emotional distress [11], and to develop specific school-based interventions incorporating interventions to decrease emotional distress and school absence [22].

Importantly, the ability to achieve greater changes in pain intensity, disability and moderate school absences via intensive inpatient treatment was also confirmed by comparing decliners of inpatient treatment who completed outpatient treatment instead to completers.

The two groups improved considerably, as reflected by almost 50% in each group achieving clinically significant changes in pain intensity, disability and approximately 70% rate of regular school attendance at the return visit, regardless of their different therapeutic choices. This finding may be indicative of additional factors influencing the decliners which may also relate to self-selection of treatment, such as a higher treatment motivation or higher family resources, which may counteract their smaller therapeutic dose. Of importance and in contrast to other health care systems, all patients in Germany have equal access to all levels of care. Hence, declining treatment was here framed as a willing decision of the child and his/her family. Results, however, also indicate greater changes in disability and moderate school absence in completers than decliners, in line with our study hypothesis. This is also reflected by the greater levels of disability and moderate school absence in decliners at the return visit. A similar added value for intensive interdisciplinary pain treatment has been shown for adult patients with chronic low back pain [23, 24]. Although these studies implemented monodisciplinary treatment approaches, such as a muscle-conditioning programme [23] or an outpatient active physiotherapy [24] as a comparison group, they all revealed a greater decrease in sick-leave days for patients who obtained intensive interdisciplinary pain treatment in accordance with the present study results.

Given that disability constitutes a prevailing maintaining factor for chronic pain, its decrease is of utmost importance and represents a number one priority treatment goal [7]. While evidence for decreases in disability via psychological pain treatment remains scarce [25], the present study indicates the potential of intensive inpatient treatment to decrease disability in highly affected children. The above-mentioned mechanism of intensive inpatient treatment may account for the greater changes in the completers.

### Limitations

The present study has several limitations. This study constitutes a naturalistic practice-based study without randomisation and lacks a control group. Any findings of significance can therefore only reflect a correlation and not a causal relationship. In addition, the study design involved a comparison of two groups - outpatients vs. inpatients - which were created among others based on baseline pain and disability and subsequently compared on change in these variables. This is a necessary approach in a naturalistic study but entails the risk of a regression-to-the-mean effect [26] and therefore a greater likelihood to find statistically significant improvements in the more severely affected inpatient group. One way to control for this effect is to compare patients with similar levels of pain intensity and disability, as was done by the comparison of decliners to completers in this study. An additional comparison of outpatients and inpatients with a comparable high level of pain intensity (of *n =* 84 outpatients and *n =* 52 inpatients) also revealed a greater reduction in disability in the inpatient-group (F_(1,134)_ = 6.80, *p =* .010). Additional ways to control for this effect which may be implemented in future studies wherever feasible are a random allocation to comparison groups [26].

Second, the described sample consisted of children with severe chronic pain conditions referred to tertiary treatment. Thus, any generalisation of these findings to less affected children may be ill-advised. Generalizability of the present findings may also be hampered due to the fact that children in the present study self-selected assignment to the two treatment options vs. treatment assignment driven by insurance.

While we did depict the return patterns of the children, assessment of the following variables was not feasible due to the naturalistic design of the study and warrants investigation in future studies: reasons for return to the treatment centre, reasons for denying intensive inpatient treatment, what treatment modules were pursued among the outpatient group and information on the transition into outpatient services. Previous studies found high rates of adherence for physical therapy and moderate adherence to begin cognitive-behavioural therapy [27].

The present outpatient treatment is of low dose. This low treatment dose at a specialized treatment centre might increase access to specialized care compared to treatments with frequent appointments. There is still a scarcity of specialized treatment centres worldwide [28] resulting in distance barriers and travel burdens for the child and his/her family [29].

Future studies are warranted comparing inpatient programs to outpatient programs that differ in length and treatment dose, into how the treatment dose might relate to treatment access of affected children, and into adherence to treatment recommendations, particularly for psychological interventions, e.g. by increasing educational efforts [27].

## Conclusion

Interdisciplinary outpatient treatment and intensive inpatient treatment are two effective forms of treatment for paediatric chronic pain that differ in their therapeutic conception and treatment intensity. Here, we replicated the effectiveness of both treatments in terms of substantial improvements in functioning and school absence within a naturalistic practice-based longitudinal study. The findings also highlight the importance of allocating children appropriately to outpatient vs. inpatient treatment. Children who denied intensive inpatient treatment and completed outpatient treatment instead achieved less improvement and were more disabled at the return visit than were the children who completed the intensive inpatient treatment. The intensive treatment also elicits greater changes in pain intensity, disability and moderate school absences, which are necessary factors for consideration among severely affected children with chronic pain. It is therefore highly relevant to enhance motivation for intensive inpatient treatment when necessary and to overcome potential barriers of children and their families. Future research is warranted into the mechanisms of change in both forms of treatment [30], into other assessment tools facilitating the allocation to the two forms of treatment [10], into therapeutic strategies to enhance the motivation for intensive inpatient treatment, and into comparisons between inpatient programs versus outpatient programs that differ in length and treatment dose, and how this might relate to treatment access.
